# Infection with hepatitis B and C virus in Europe: a systematic review of prevalence and cost-effectiveness of screening

**DOI:** 10.1186/1471-2334-13-181

**Published:** 2013-04-18

**Authors:** Susan JM Hahné, Irene K Veldhuijzen, Lucas Wiessing, Tek-Ang Lim, Mika Salminen, Marita van de Laar

**Affiliations:** 1 Centre for Infectious Disease Control, National Institute for Public Health and the Environment (RIVM), PO Box 1, Bilthoven, 3720 BA, The Netherlands; 2 Municipal Public Health Service Rotterdam-Rijnmond, Rotterdam, PO Box 70032, 3000 LP, The Netherlands; 3 European Centre for Disease Prevention and Control (ECDC), Tomtebodavägen 11a, Stockholm, 171 83, Sweden; 4 European Monitoring Centre for Drugs and Drug Addiction (EMCDDA), Cais do Sodré, Lisbon, 1249-289, Portugal

**Keywords:** Hepatitis B virus, Hepatitis C virus, Europe, Prevalence, HBsAg, Anti-HCV-Ab, Cost-effectiveness analyses

## Abstract

**Background:**

Treatment for chronic hepatitis B virus (HBV) and hepatitis C virus (HCV) infection is improving but not benefiting individuals unaware to be infected. To inform screening policies we assessed (1) the hepatitis B surface antigen (HBsAg) and anti-hepatitis C virus antibody (anti-HCV-Ab) prevalence for 34 European countries; and (2) the cost-effectiveness of screening for chronic HBV and HCV infection.

**Methods:**

We searched peer-reviewed literature for data on HBsAg and anti-HCV-Ab prevalence and cost-effectiveness of screening of the general population and five subgroups, and used data for people who inject drugs (PWID) and blood donors from two European organizations. Of 1759 and 468 papers found in the prevalence and cost-effectiveness searches respectively, we included 124 and 29 papers after assessing their quality. We used decision rules to calculate weighted prevalence estimates by country.

**Results:**

The HBsAg and anti-HCV-Ab prevalence in the general population ranged from 0.1%-5.6% and 0.4%-5.2% respectively, by country. For PWID, men who have sex with men and migrants, the prevalence of HBsAg and anti-HCV-Ab was higher than the prevalence in the general population in all but 3 countries. There is evidence that HCV screening of PWID and HBsAg screening of pregnant women and migrants is cost-effective.

**Conclusion:**

The prevalence of chronic HBV and HCV infection varies widely between European countries. Anti-HCV-Ab screening of PWID and HBsAg screening of pregnant women and migrants have European public health priority. Cost-effectiveness analyses may need to take effect of antiviral treatment on preventing HBV and HCV transmission into account.

## Background

Hepatitis B and C virus (HBV and HCV) infect the liver and can lead to a broad spectrum of disease outcomes. Between 15% and 40% of those chronically infected with HBV or HCV will in their lifetimes develop serious liver disease due to cirrhosis and/or hepatocellular cancer (HCC) [1,2]. People with chronic infection with HBV or HCV can remain infectious to others. Both HBV and HCV are widely present with broad variation in prevalence by country [3]. Worldwide, between 350 to 400 million people are infected with HBV, accounting for 1 million deaths per year [4,5]. Between 130 and 170 million people are infected with HCV, causing over 350,000 deaths per year [6].

A safe and effective vaccine for HBV has been available since 1982, whereas no vaccine for HCV exists [7]. Treatment options are advancing rapidly, and several new antiviral drugs have become available in the past decade. Evidence is accumulating that these therapies provide a cost-effective means to reduce the morbidity and mortality associated with chronic infection with HBV and HCV [8-10]. European treatment guidelines for chronic HBV and HCV infection are available [11,12]. In addition to improving the outcome of chronic hepatitis, antiviral treatment is likely to reduce transmission by reducing the viral load and therefore infectivity of chronic carriers, similar to what has been documented for HIV [13-15]. For HBV, vaccination of susceptible contacts of identified carriers can prevent new infections. Nonpharmaceutical interventions, such as the advice to limit alcohol intake and cease smoking, can improve outcomes for people living with chronic viral hepatitis [16,17].

Since the acquisition of HBV and HCV is often asymptomatic or subclinical, and sequelae take several decades to develop, between 40% and 80% of people with chronic hepatitis are unaware of their infection [18-25]. Therefore, screening programmes for chronic HBV and HCV infection have the potential to contribute considerably to primary and secondary prevention of these infections. However, existing HBV and HCV screening programmes in Europe stem from an era when treatment options for chronic viral hepatitis were limited. Hence they are mainly aimed at primary prevention, targeting blood donors, pregnant women, and behavioral high-risk groups [26]. Now that secondary prevention of HBV and HCV is possible, there is an urgent need to identify chronic carriers who may benefit from treatment.

For policy development in this area data on the size and characteristics of the population with chronic hepatitis and the evidence for cost-effectiveness of screening are needed. The most recent HBsAg prevalence review including data on European countries was from 2004, and reported findings from only 11 European countries [27]. For HCV, a review of the burden of disease in Europe was published in 2009 [28]. In this review, however expert opinion was a main source of data, which makes the validity of conclusions difficult to ascertain. Esteban reviewed the HCV prevalence in Europe in 2008 [29], but studies on blood donors were included as estimates for the general population. Regarding the cost-effectiveness of screening for HCV, an earlier review included studies published up to March 2007 [30]. It concluded that HCV screening of both former and current PWID was cost-effective. Systematic reviews of cost-effectiveness of screening for HBV infections have not been published.

To address the missing information we performed a systematic literature review of the prevalence of hepatitis C virus antibodies (anti-HCV-Ab) and hepatitis B surface antigen (HBsAg) in the general population and five population subgroups (pregnant women, first-time blood donors, people who inject drugs [PWID], men who have sex with men [MSM], and migrants) for 34 European countries.^a^ We subsequently used our prevalence estimates to assess the total number of people living with chronic HBV and HCV infection by country. To further build the evidence base for secondary prevention we also performed a systematic review of the cost-effectiveness of HBsAg and anti-HCV screening of the general population and population sub-groups.

## Methods

### Hepatitis B and C prevalence

To find studies that describe prevalence of HBsAg and anti-HCV-Ab (the serological markers used as proxies for chronic infection in this study) we searched Medline, Embase, and SciSearch for English-language, peer-reviewed literature published between 1 January 2000 and 27 July 2009. Reference lists of included studies were hand searched. Studies were eligible for inclusion in the review if they reported the anti-HCV-Ab and/or HBsAg prevalence in the 34 countries included in our review, in the general population or among pregnant women, first-time blood donors, MSM, or migrants. Studies that reported on children only were not included. We only used the most recent estimate when more than 1 regional estimate was available based on studies performed 5 or more years apart. When several estimates were available for a specific country, an average weighted by study size was calculated. For first-time blood donors, we used data from a report for the Council of Europe in addition to data from the published literature [31]. Anti-HCV-Ab and HBsAg prevalence estimates among PWID were obtained from 2 sources: the European Monitoring Centre for Drugs and Drug Addiction (EMCDDA) [32] and from a recent review on HBV and HCV prevalence among PWID [33]. The source with the most recent national prevalence estimate was used. We excluded estimates from studies performed before 2000, with fewer than 50 participants, and where injecting drug use status was unknown.

### Costs effectiveness of screening for hepatitis B and C

For the systematic literature review of the evidence for cost-effectiveness of screening for chronic HBV and/or HCV infection we searched Medline, Scopus, and the NHS Economic Evaluation Database (EED) for studies published in the English-language, peer-reviewed literature between 1 January 2000 and 31 December 2012. Studies reporting only on screening of transfusion recipients and/or of patients treated by infected health care workers (‘look-back studies’) were excluded. Studies were only eligible when reporting estimated costs per additional chronic infection identified and/or costs per life year (LY) gained (quality or disability adjusted). Cost estimates were converted into 2010 Euros using information from Eurostat and OECD [34,35].

#### Data extraction

For both systematic literature searches, data were extracted using a data-extraction form by two authors (SH and IV). For the prevalence search, the form included year, country population of the study, the sampling method, laboratory test used, participation rate, number of participants, and HBsAg and anti-HCV-Ab results. For the cost-effectiveness search, the form included year and country of study, target population for screening, screening scenario, type of model used, outcome measure(s) used, monetary value and year, discounting percentage (costs/effects), results, and conclusions. The quality of the prevalence studies was assessed by reviewing the representativeness of sampling (eg, random vs convenience sampling) and, for the general population, whether estimates were standardized by age and sex.

Prevalence estimates were summarized by country. When multiple general population prevalence estimates were available for one country, we used the estimate that was most representative for the entire country regarding demographic coverage. In case multiple representative general population prevalence estimates for one country were found, the average prevalence was calculated weighted by study size. Where estimates for 3 or more regions in a country were available, regional estimates were presented only when the difference between regions was more than 0.5%. Countries were grouped into low, intermediate, and high HBsAg and anti-HCV-Ab prevalence using cut offs of ≤ 1%, > 1% to ≤ 2%, and > 2%. On the basis of prevalence estimates in the general population for infection with HBV and HCV and 2009 population size [36], we estimated the total number of people in that country who would likely test positive for HBsAg or anti-HCV-Ab. Search terms used for both searches are available in Additional file 1. The methods of our systematic literature reviews and their reporting are consistent with those recommended by the PRISMA statement and specified in advance in a protocol that is available from the corresponding author on request [37].

## Results

### Seroprevalence of hepatitis B and C

The search for data on the HBsAg and/or anti-HCV-Ab prevalence in the general population and 5 subgroups identified 1759 citations, from which the full-text publication of 236 (13%) was retrieved. From the reference lists of included studies, an additional 8 potentially relevant citations were identified. After review of the full text of these 244 papers, 53 publications were considered not relevant. Furthermore, 67 publications on PWID were excluded, since prevalence estimates from PWID were obtained from the EMCDDA and a recent literature review [33]. Finally, 124 publications were included in the review of prevalence data, with 81 publications used for the prevalence estimate for the general population or population subgroups. A flow diagram depicting the inclusion of studies is available in the Additional file 2.

#### General population

**Figure 1:**
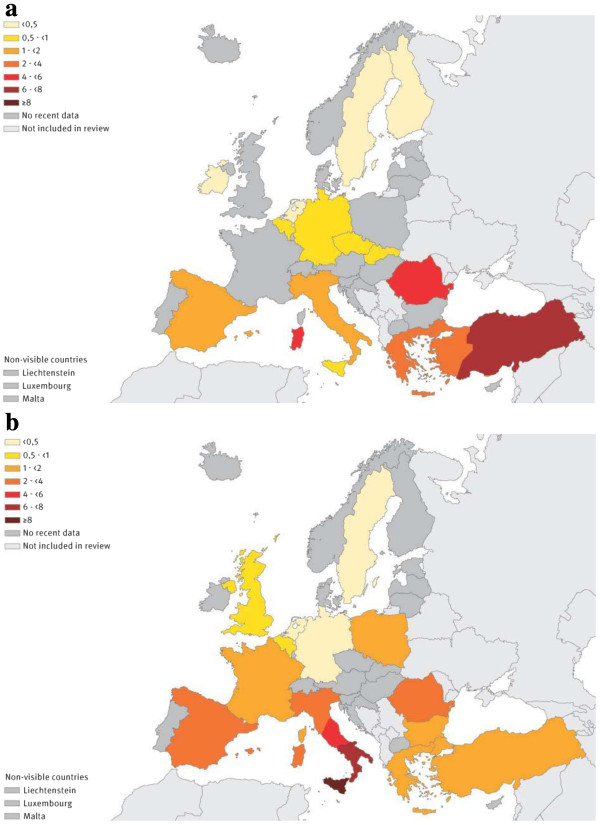
**Hepatitis B and hepatitis C prevalence (%) in the general population by country, Europe, 2000–2009. a.** Hepatitis B surface antigen (HBsAg) prevalence (%). **b.** Hepatitis C (anti-HCV-antibody) prevalence (%).

HBsAg general population prevalence estimates were found for 13 of the 34 countries in our review, ranging from 0.1% to 5.6% by country (Figure 1a, Table 1). The estimated number of people with chronic HBV infection ranged from 3,718,889 in Turkey to 4,466 in Ireland (Table 1). Prevalence estimates of anti-HCV-Ab in the general population were found for 13 of the 34 countries in our review, ranging from 0.4% to 5.2% by country (Figure 1b, Table 2). The estimated number of people who were anti-HCV-Ab positive ranged from 3,122,779 in Italy to 37,025 in Sweden (Table 2). For only a minority of countries (9/34) information was available on both estimates. Countries in the north-western part of Europe had a low prevalence for both infections whilst those in the south and south-east had an intermediate to high prevalence (Additional file 3: Figure S2).

**Table 1:** Estimates of general population HBsAg prevalence, and number of HBsAg positive people in the general population, by country, Europe, 2000-2009

Country* (Reference)	**Period**	**Area**	**Region**	**Sampling**	**N**	**%**	(95% CI)**	**Remarks**	**Population size **[36]	**Number of HBsAg-positive inhabitants**
Belgium [91]	2003	regional	Flanders	random	1.834	0,7	(0,5-0,8) &	Oral fluid	10.754.528	75.282
Czech Republic [92]	2001	nationwide	-	random	2.658	0,6	(0,3-1,0) $	Standardized	10.467.542	62.805
Finland [93]	1997-1998	nationwide	-	residual	3.083	0,2	(0,1-0,4) $		5.326.314	10.653
Germany [94]	1993-1996	nationwide	-	random	5.305	0,6	(0,4-0,8) &		82.050.000	492.300
Germany [95]	1998	nationwide	-	random	6.748	0,6	(0,4-0,8) &			
Greece [96]	1997-1998	regional	Peloponnesos	random	1.500	2,1	(1,5-3,0) &		11.257.285	236.403
Ireland [93]	2003	nationwide	-	residual	2.535	0,1	(0,0-0,3) $		4.465.540	4.466
Italy [93]	1996	nationwide	-	residual	3.522	0,6	(0,4-1,0) $		60.053.442	840.748
Italy [74]	2002	regional	North	convenience	956	1,0	(0,5-1,9) $			
Italy [97]	1997	regional	Central	random	250	1,2	(0,3-3,5) $			
Italy [98]	1997	regional	South	random	488	0,2	(0,0-3,5) $			
Italy [99]	2002-2003	regional	South	random	1.645	1,8	(0,4-1,2) &			
Italy [100]	1994-1994	regional	Sardinia	convenience	3.324	4,3	(3,6-5,1) $			
Italy [101]	1999-2000	regional	Sicily	random	721	0,7	(0,2-1,6) $			
Netherlands [93]	1995-1996	nationwide	-	random	6.750	0,1	(0,0-0,2) $		16.486.587	16.487
Romania [93]	2002	nationwide	-	residual	1.259	5,6	(4,4-7,0) $		21.498.616	1.203.922
Slovakia [93]	2002	nationwide	-	random	3.569	0,6	(0,4-0,9) $		5.412.254	32.474
Spain [102]	1996	regional	Catalonia	random	2.142	1,2	(0,7-1,7) &	Standardized	45.828.172	458.282
Spain [103]	2002	regional	Catalonia	random	2.620	0,7	(0,4-1,0) &			
Sweden [104]	1991-1994	regional	Malmö	random	5.533	0,2	(0,1-0,4) $		9.256.347	18.513
Turkey [105]	2006-2007	regional	West	random	2.852	2,5	(2,0-3,1) $		71.517.100	3.718.889
Turkey [106]	2002-2004	regional	Central	convenience	1.320	6,6	(5,3-8,1) $			
Turkey [107]	1996	regional	Central	convenience	571	6,7	(4,8-9,1) $			
Turkey [108]	Not reported	regional	Central	random	1.095	5,5	(4,2-7,0) $			
Turkey [109]	1997-1999	regional	East	convenience	400	9,0	(6,4-12,2) $	32-year-olds		
Turkey [110]	2003	regional	East	random	2.888	7,0	(6,1-8,0) $			

* No estimate of HBsAg general population prevalence was found for Austria, Bulgaria, Croatia, Denmark, Estonia, Former Yugoslav Republic of Macedonia, France, Hungary, Iceland, Latvia, Liechtenstein, Lithuania, Luxembourg, Malta, Norway, Poland, Portugal, Slovenia, Switzerland and the United Kingdom.

** CI Confidence interval.

& CI provided in the original paper.

$ CI estimated by the exact method.

**Table 2:** Estimates of general population anti-HCV-Ab prevalence, and number of anti-HCV positive people in the general population, by country, Europe, 2000-2009

Country* (Reference)	**Period**	**Area**	**Region**	**Sampling**	**N**	**%**	95% CI**	**Remarks**	**Population size **[36]	**Number of inhabitants who are anti-HCV-Ab positive**
Belgium [91]	2003	regional	Flanders	random	1.834	0,1	(0.1-0.4) &	Oral fluid	10.754.528	64.527
Belgium [111]	1993-1994	regional	Flanders	random	4.055	0,9	(0.5-1.1) &			
Bulgaria [112]	1999-2000	regional	South-Central	convenience	2.211	1,3	(1.2-1.4) &	Standardized	7.606.551	98.885
Czech Republic [92]	2001	nationwide	-	random	2.658	0,2	(0.1-0.4)$		10.467.542	20.935
France [113]	1997	regional	South	convenience	11.804	1,3	(1.1-1.5) &		64.351.000	836563
Germany [95]	1998	nationwide	-	random	6.748	0,4	(0.2-0.5) &		82.050.000	328200
Greece [96]	1997-1998	regional	Peloponnesos	random	1.500	0,5	(0.2-1.1) &		11.257.285	112.573
Greece [114]	1997	regional	Zakinthos	random	718	1,3	(0.6-2.4) $			
Italy [74]	2002	regional	North	convenience	956	2,6	(1.7-3.8) $			
Italy [115]	1994-1995	regional	North	convenience	2.154	3,3	(2.6-4.1) &			
Italy [116]	Not reported	regional	North	convenience	4.820	2,4	(2.0-2.8) &			
Italy [117]	Not reported	regional	Central	convenience	300	16,3	(12.0-20.6) &			
Italy [97]	1997	regional	Central	random	250	22,4	(20.8-24.1) &			
Italy [98]	Not reported	regional	South	random	488	16,2	(13.0-19.8) $			
Italy [118]	2000-2002	regional	South	convenience	2.753	7,9	(6.9-9.0) $			
Italy [99]	2002-2003	regional	South	random	1.645	6,5	(5.3-7.7) &			
Italy [100]	1994-1995	regional	Sardinia	convenience	3.324	3,2	(2,6-3,8) $			
Italy [101]	1999-2000	regional	Sicily	random	721	10,4	(8,2-12,9) $			
Netherlands [41]	2004	regional	Amsterdam	random	1.364	0,6	(0.1-1.1) &	Standardized	16.486.587	65.946
Netherlands [119]	2006	regional	East	convenience	2.200	0,2	(0.1-0.5) $			
Poland [120]	1999	regional	North	convenience	2.561	1,9	(1.4-2.5) $		38.135.876	724.582
Romania [121]	2006-2008	nationwide	-	random	8.039	3,5	(3.1-3.9) &		21.498.616	752.452
Spain [122]	1996	regional	Catalonia	random	2.142	2,5	(1.8-3.2) &	Standardized	45.828.172	916.563
Spain [123]	1997-1998	regional	North	random	1.170	1,6	(1.0-2.6) &			
Sweden [104]	1991-1994	regional	Malmö	random	5.533	0,4	(0,3-0,6) $		9.256.347	37.025
Turkey [105]	2006-2007	regional	South West	random	2.852	1,0	(0,7-1,4) $		71.517.100	1.072.757
Turkey [106]	2002-2004	regional	Central	convenience	1.320	2,2	(1,5-3,1) $			
Turkey [108]	Not reported	regional	Central	random	1.095	2,1	(1,3-3,1) $			
United Kingdom [124]	1996	regional	England&Wales	residual	6.401	0,7	(0,1-0,5) $		61634599	431442

* No estimate of anti-HCV general population prevalence was found for Austria, Croatia, Cyprus, Denmark, Estonia, Finland, Former Yugoslav Republic of Macedonia, Hungary, Iceland, Ireland, Latvia, Liechtenstein, Lithuania, Luxembourg, Malta, Norway, Portugal, Slovakia, Slovenia and Switzerland.

** CI Confidence interval.

& CI provided in the original paper.

$ CI estimated by the exact method.

#### Blood donors

HBsAg prevalence estimates for first-time blood donors were found for 24 countries, ranging from 0.0% to 5.2% (Additional file 3: Figure S3.1a, Additional file 4: Table S3.1.a). Anti-HCV-Ab prevalence estimates for first- time blood donors were available for 23 countries, ranging from 0.02% to 3.3% (Additional file 3: Figure S3.1b, Additional file 4: Table S3.1.b). The prevalence of HBsAg and anti-HCV-Ab in first-time blood donors was on average respectively 3 and 4 times lower than the corresponding prevalence for the general population in countries that had both estimates available (12 countries for HBsAg and 11 for anti-HCV-Ab).

#### Pregnant women

Estimates of antenatal HBsAg prevalence were found for 11 countries, ranging from 0.1% to 4.4% (Additional file 3: Figure S3.2a, Additional file 4: Table S3.2.a). Estimates of antenatal anti-HCV-Ab prevalence were found for 6 countries, ranging from 0% to 1.7% (Additional file 3: Figure S3.2b, Additional file 4: Table S3.2.b). The antenatal HBsAg prevalence was on average 3 times higher than the general population prevalence in 6 of the 7 countries that had both estimates available. The country where it was lower was Spain (based on regional data from Catalonia), likely reflecting the effect of the HBV vaccination programme for adolescents. In Italy and the United Kingdom, the antenatal anti-HCV-Ab prevalence was lower than the general population prevalence. In Germany and Greece, it was higher.

#### PWID

An estimate of HBsAg prevalence in PWID was available for 21 of the 34 countries in this review, ranging from 0% to 21.3% (Additional file 3: Figure S3.3a, Additional file 4: Table S3.3.a). An estimate of anti-HCV-Ab prevalence in PWID was available for 29 of the 34 countries, ranging from 5.3% to 90% (Additional file 3: Figure S3.3b, Additional file 4: Table S3.3.b). The HBsAg prevalence in PWID was on average 9 times higher than that in the general population (in 6 of the 8 countries that had both estimates available). In Romania and Ireland, the general population HBsAg estimate was higher. The estimate of anti-HCV-Ab prevalence in PWID was on average 47 times higher than that in the general population (in 13 countries that had both estimates available).

#### Migrants

Estimates of HBsAg prevalence in migrants were found for 5 countries. The HBsAg prevalence in migrants ranged from 1.0% to 15.4% (Additional file 4: Table S3.4.a). Estimates of anti-HCV-Ab prevalence in migrants were found for 5 countries, ranging from 0% to 23.4% (Additional file 4: Table S3.4.b). The estimate of HBsAg and anti-HCV-Ab prevalence in migrants was on average respectively 6 and 2 times higher than that in the general population in all countries that had both estimates available (4 countries for HBsAg and 4 for anti-HCV-Ab), except for Italy, where the estimate of anti-HCV-Ab prevalence in migrants was lower than that in the general population.

#### MSM

Estimates of HBsAg prevalence for MSM were found for 3 countries, ranging from <1% to 4% [38-41]. Estimates of anti-HCV-Ab prevalence among MSM were available for 3 countries, ranging from 0.07% to 2.9% [41-43]. The HBsAg and anti-HCV-Ab prevalence in MSM was on average respectively 22 and 3 times higher than that for the general population in all countries that had both estimates available (2 countries for HBsAg and 1 for anti-HCV-Ab).

### Cost-effectiveness of HBV and HCV screening

The search for evidence on cost-effectiveness of HBV and/or HCV screening identified 468 publications. We retrieved the full text for 41 publications (9%). From the reference lists of included studies 3 additional potentially relevant citations were identified. Of these 44 papers, 13 were considered not relevant following full text review. Two additional publications were excluded [44,45] because they reported on data that were more extensively presented in a third publication [46]. Finally, 29 publications were included in the review of cost-effectiveness of screening (flow chart Additional file 2). No paper studied combined screening for HBV and HCV. Of the 29 papers, 23 used a Markov model (21 with hypothetical data and 2 presented actual screening results). The remaining 6 studies did not use a model and presented costs per case identified or cost per infection prevented. None of the studies included dynamic modeling to take into account effects of reducing transmission by lowering viral load through treatment, behavior change, or HBV vaccination.

**Table 3:** Publications included in the cost-effectiveness review (n = 29)

**First Author, year, reference**	**Target group**	**Setting**	**Country**	**Infection**	**Model**	**Data**	**Indicator^**	Result*	**Year of monetary value #**	**Euro in 2010**	**Cost-effective?**
Thomas, 1990 [58]	Pregnant women	Antenatal care	Australia	HBV	None	Actual screening	Cost per case detected	$354 (AU)	1988	€ 379	Yes
Audet, 1991 [57]	Pregnant women	Antenatal care	Canada	HBV	None	Hypothetical cohort	Cost per case detected/infant carrier prevented	$1.693/$8.915 (CA)	1988	€ 1.799-€9.475	Yes, probably
Tormans, 1993 [54]	Pregnant women	Antenatal care	Belgium	HBV	Markov	Hypothetical cohort	Cost per LY gained	BEF 583.581	1991	€ 22.095	Yes
Dwyer, 1996 [56]	Pregnant women	Antenatal care	UK	HBV	Markov	Hypothetical cohort	Cost per carrier prevented/LY gained	£2.437/£16.450	Not mentioned (1996)	€ 3.879 -€26.181	Yes
Jordan, 1997 [55]	Pregnant women	Antenatal care	Britain	HBV	Markov	Hypothetical cohort	Cost per LY gained	₤1.300	Not mentioned (1997)	€ 2.032	Yes
Plunkett, 2005 [59]	Pregnant women	Antenatal care	USA	HCV	Markov	Hypothetical cohort	Cost per QALY	No screening dominant	2003	n.a.	No
Eckman, 2011 [47]	General population (35 year old males)	Primary care	USA	HBV	Markov	Hypothetical cohort	Cost per QALY	$29.232 (US)	2008	€ 23.966	Yes
Singer, 2001 [49]	General population	Not specified	USA	HCV	Markov	Hypothetical cohort	Cost per QALY	No screening dominant	2001	n.a.	No
Nakamura, 2008 [50]	General population & risk groups	Not specified	Japan	HCV	Markov	Actual screening	Cost per LY gained	$848 - $4.825 (US)	2007	€ 726-€4.130	Yes
Loubiere, 2003 [48]	General population, IDUs & other risk groups	Not specified	France	HCV	Markov	Hypothetical cohort	Cost per LY gained	$4.513 (US) (IDUs)/$5.821 (gen pop)	1998	€ 4.856/€6.263	Yes
Coffin, 2012 [51]	General population (20–69 y.o. and 1945–1965)	Not specified	USA	HCV	Markov	Hypothetical cohort	Cost per QALY	$7.900-$5.400 (US)	2010	€ 6.376-€4.358	Yes
McGarry, 2012 [52]	General population (born 1946–1970)	Not specified	USA	HCV	Markov	Hypothetical cohort	Cost per QALY	$37.720 (US)	2010	€ 30.444	Yes
Rein, 2012 [53]	General population (born 1945–1965)	Not specified	USA	HCV	Markov	Hypothetical cohort	Cost per QALY	$15.700 (US)	2009	€ 12.976	Yes
Ruggeri, 2011 [73]	High risk groups	Not specified	Italy	HBV	Markov	Hypothetical cohort	Cost per QALY	€18.255 (IT)	2004	€ 52.885	Yes
Hutton, 2007 [67]	Migrants	Not specified	USA	HBV	Markov	Hypothetical cohort	Cost per QALY	$36.088 (US)	2006	€ 31.692	Yes
Veldhuijzen, 2010 [68]	Migrants	Population based	Netherlands	HBV	Markov	Hypothetical cohort	Cost per QALY	€8.966 (Nl)	2009	€ 8.694	Yes
Rein, 2011 [69]	Migrants	5 settings compared	USA	HBV	None	Actual screening	Cost per case detected	$609-$4.657 (US)	2008	€499-€3.818	Yes
Wong, 2011 [70]	Migrants	Not specified	Canada	HBV	Markov	Hypothetical cohort	Cost per QALY	$69.209 (CA)	2008	€46.260	Yes, moderately
Leal, 1999 [60]	IDUs	Drug services	UK	HCV	Markov	Hypothetical cohort	Cost per QALY	£9.300	1997	€ 14.540	Yes
Castelnuovo, 2006 [61]	IDUs	Various	UK	HCV	Markov	Hypothetical cohort	Cost per QALY	£15.493-£20.083	2004	€ 22.172-€28.741	Yes
Thompson Coon, 2006 [62]	IDUs	Primary care	UK	HCV	Markov	Hypothetical cohort	Cost per QALY	£16.493	2002/2003	€ 24.245	Yes
Kerr, 2009 [43]	IDUs & MSM	STD clinic	Scotland	HCV	None	Actual screening	Cost per case detected	£ 170 (IDU)/£15.000 (MSM)	Not mentioned (2009)	€ 215-€18.975	Yes (IDU), No (MSM)
Josset, 2004 [63]	IDUs & other risk groups	Primary care	France	HCV	None	Actual screening	Cost per case detected	not reported	Not mentioned	n.a.	Not stated
Stein, 2004 [44]	IDUs & other risk groups	STD clinic/drug services	UK	HCV	Markov	Hypothetical cohort	Cost per QALY	₤28.120 (IDUs)/₤84.570 (GUM)	2001	€ 41.874-€125.933	Yes (IDUs)/No (GUM)
Honeycutt, 2007 [64]	IDUs & other risk groups	STD clinic	USA	HCV	None	Hypothetical cohort	Cost per case detected	$54 (US)	2006	€ 47	Yes
Tramarin, 2008 [65]	IDUs & other risk groups	Not specified	Italy	HCV	Markov	Hypothetical cohort	Cost per QALY	-€3.132 (IT)	2007	-€ 3.328	Yes
Helsper, 2012 [66]	IDUs & other risk groups incl migrants	Primary care/drug services	Netherlands	HCV	Markov	Actual screening	Cost per QALY	€7.321 (NL)	2007	€ 7.327	Yes
Sutton, 2006 [71]	Prisoners	Prison	UK	HCV	Markov	Hypothetical cohort	Cost per case detected	£2,102 - £3,107	2004	€ 3.008-€4.446	Yes
Sutton, 2008 [72]	Prisoners	Prison	UK	HCV	Markov	Hypothetical cohort	Cost per QALY	£54.852	2004	€ 78.498	No

* Results of most favorable scenario reported here.

# Where the paper did not quote year of monetary value the year of publication was used to convert the results into 2010 Euros [34].

n.a. Not available.

^ *QALY*, Quality adjusted life year; *LY*, Life year.

#### General population screening

One economic analysis of HBsAg screening of the general population (considering in the base case 35 year old males with a 2% prevalence) found this was cost-effective (incremental cost-effectiveness ratio (ICER) €23.966/quality adjusted life year (QALY)) [47]. Six studies reported on HCV screening and subsequent treatment of the general population [48-53]. Two of these reported costs per LY gained, both concluding HCV screening of the general population was cost-effective [48,50]. Four studies, all from the USA, assessed general population HCV screening by estimating cost per QALY gained [49,51-53]. All studies except one [49] concluded general population HCV screening of adults was cost-effective.

#### Antenatal screening

Five economic analyses reported on antenatal HBsAg screening [54-58], presenting estimated costs per LY gained [54-56], costs per case detected and per infant carrier prevented [57] and costs per case detected [58]. The 3 studies presenting costs per LY gained studied the scenario of universal screening of all pregnant women, with vaccination of infants born to HBsAg positive mothers. Studies were published between 1993 and 1997, and none considered antiviral treatment. ICERs ranged from €2,032 to €26,181 per LY gained. All studies concluded that universal antenatal HBV screening is cost-effective considering the respective thresholds used. One economic analysis of antenatal HCV screening was found, which considered universal antenatal HCV screening and treatment of HCV infection with or without elective cesarean delivery [59]. Neither of these scenarios was considered cost-effective.

#### PWID

No economic analysis of HBsAg screening of PWID was found. Ten studies reported on cost-effectiveness of HCV screening and treatment of PWID [43,46,48,60-66]. Seven of these reported estimated costs per QALY. These studies varied widely, including different screening settings, treatments considered, and discount rates. Nevertheless, all 7 studies concluded that HCV screening of PWID was likely to be cost-effective considering the respective thresholds used, with ICERs ranging from ─ €3.328 to €41,874 per QALY.

#### Migrants

Four economic analyses of screening migrants for HBsAg were found [67-70]. One of these compared 4 community outreach screening programs by assessing cost per person screened and cost per HBsAg positive individuals identified, concluding that screening in outpatient settings was the most cost-effective but reached the lowest number of participants [69]. The 3 other studies assessing cost per QALY all concluded migrant screening was cost-effective, with ICERs ranging from €8.694 [68] to €46.260 [70]. One economic analysis of HCV screening of migrants was found [66]. In this study, the target group for screening included migrants from countries with a HCV prevalence >10%, as well as from other population subgroups. Separate estimates of cost-effectiveness of screening migrants were, however, not presented.

#### MSM

We found no economic analysis of HBsAg screening of MSM. One economic analysis of HCV screening of MSM at sexually transmitted disease (STD) clinics concluded that HCV screening of MSM in this setting was not cost-effective [43].

#### STD-clinic attenders

We did not find any economic analyses of HBsAg screening of STD-clinic attendees, but 2 of HCV screening of STD-clinic attendees [46,64]. Universal screening and treatment of UK STD-clinic attendees was assessed as not cost-effective (ICER €125,933/QALY) [46,64]. Among STD-clinic attendees in the US, HCV screening of non-PWID was only cost-effective when restricted to men with >100 lifetime sex partners [64].

#### Prisoners

No economic analyses of HBsAg screening of prisoners were found. Regarding HCV screening of prisoners, we found two studies both from England and Wales [71,72]. The first study found that asking prisoners about their HCV and injecting status prior to laboratory testing can considerably reduce the cost per case detected [71]. The second paper found that HCV screening and treatment of prisoners was not cost-effective (cost per QALY €78,498) [72].

#### Other high-risk groups

One study assessed HBsAg screening of high-risk groups currently recommended for screening in Italy as cost-effective [73]. However, no costs for the screening programme were taken into account and compliance was set at an unrealistic 100%. Five economic analyses of HCV screening of other population subgroups were found [48,50,63,65,66,74]. Both studies that considered screening programmes targeting several population subgroups concluded that the specific programmes considered were potentially cost-effective [50,66]. Josset et al. reported estimated costs per positive test result for 6 screening scenarios, which varied regarding population subgroups targeted [63]. Analyses of HCV screening of people transfused before 1991 in France and of people with a history of surgery in Italy both concluded this was not cost-effective [48,65].

## Discussion and conclusions

### Prevalence of hepatitis B and C

The general population prevalence of chronic HBV and HCV infection varies widely between European countries, with those in the south and east of the European Union and in Turkey having a much higher prevalence than those in northwestern Europe. Among countries for which data were available for both infections, Romania stands out, with high prevalence for both HBV and HCV. In contrast, Belgium, Sweden, Germany, and The Netherlands have low-population prevalence for both infections. Results from a study published after our literature search was completed suggest that France also belongs to this latter category [24]. A recent systematic review of chronic HBV prevalence in Turkey was consistent with our findings reporting a similar west to east gradient [75]. For HCV, Italy had the highest estimated population prevalence, much higher than its estimated HBV prevalence. Epidemiologic and phylogenetic assessments suggest that this may have been caused by a period of frequent iatrogenic transmission that took place around the 1950s [76]. Without screening and early treatment, these infections will lead to a considerable disease burden and many deaths due to liver disease in the coming decades. Given that HBV and HCV disproportionately affect disadvantaged groups and less affluent countries in Europe, these infections will also contribute to increasing inequalities in health.

For the majority of countries, data on the general population prevalence of HBV or HCV were lacking. Availability of sufficiently recent estimates is necessary to be able to prioritize primary and secondary prevention of HBV and HCV among other public health interventions, to evaluate control measures, and for health care planning. Estimates of prevalence obtained from blood-donor and antenatal screening were found to differ substantially from general population estimates. Within countries, the prevalence of HBsAg and anti-HCV-Ab among PWID, MSM, and migrants was much higher than the corresponding prevalence in the general population, with only a few exceptions. The higher HBsAg prevalence among migrants was confirmed by a recent systematic review [77]. Of the high-risk groups considered, PWID had by far the highest prevalence, particularly for HCV.

#### Cost-effectiveness of screening for hepatitis B and C

The search for evidence on cost-effectiveness of screening was consistent with the prevalence review: For all three population subgroups with evidence of increased HBV and HCV prevalence compared to the general population (PWID, MSM and migrants) economic analyses of screening were found. This resulted in evidence that HCV screening of PWID and HBsAg screening of pregnant women and migrants are cost-effective interventions to reduce the burden of disease due to viral hepatitis. HCV screening of pregnant women and comprehensive screening of all STD-clinic attendees is probably not cost-effective, although there may be exceptions for some specific local or subpopulation conditions. General population screening for HCV was found cost-effective in the US ‘baby-boom generation’ (born 1945–1965). For other programs, including HBV screening of PWID, HCV screening migrants, HBV and HCV screening of prisoners and MSM, the evidence found in this systematic review was insufficient to draw conclusions.

#### Screening of PWID

The strongest evidence regarding cost-effectiveness was available for HCV screening of PWID. The wide range in ICER estimates may be partly explained by differing definitions of PWID, whereby some studies may include former PWID. It is unclear to what extent PWID in Europe are offered HCV screening and are successfully referred once found to be positive. HCV screening programmes for PWID exist in only 16 of the 29 European Union/European Economic Area countries reviewed in 2009 [26] whereby testing coverage and referral to treatment often remain poor [78]. On the other hand, several countries without screening programmes report adequate HCV testing of PWID [79]. This apparent discrepancy may be explained by a lack of definition of what a screening programme entails and/or the possibility that the 2009 review has missed screening programmes of PWID. Nevertheless, optimizing implementation of testing guidelines for PWID and monitoring of this group are among the highest priorities [80,81].

#### Antenatal screening

Regarding HBsAg screening of pregnant women it is likely that it would be even more economically favourable if antiviral treatment of the mother was considered. European countries that currently have selective or no antenatal HBsAg screening programmes, including Bulgaria, Lithuania, Luxembourg, Romania, and Norway, should consider implementing universal antenatal screening [26]. This holds even if these countries have universal infant HBV-vaccination programme with an at-birth dose of vaccine, since prevention of perinatal HBV transmission requires the first dose of vaccine to be given within 24 hours and a very high uptake of vaccination. In addition, providing hepatitis B immunoglobulin is of additional effectiveness [82,83].

#### Migrant screening

The four publications examining HBsAg screening of migrants born in endemic countries (HBsAg prevalence ≥ 2%) suggest this is cost-effective. Main determinants of ICER were the proportion of eligible people starting treatment, disease progression rates with and without treatment, and costs of treatment [67,68]. Further research should focus on these areas of uncertainty, as well as on how to optimize participation in screening and referral pathways [69,84]. Given that HCV could be tested using the same blood sample and that migrants generally have higher HCV prevalence than the indigenous population in European countries, an economic assessment of combined HBV/HCV screening for migrants is a priority [85,86].

#### General population screening

The only study found that considered general population screening for HBsAg [47], suggested this would be cost-effective in populations with a prevalence above 0.3%. This includes nearly all European countries. However, the study considered only men, included no costs for the screening programme (except for a blood test and consultation) and made unrealistic assumptions regarding compliance with treatment. The evidence for general population HBsAg screening can therefore be considered weak. Regarding general population screening for anti-HCV recent studies mainly from the USA suggest this is cost-effective, particularly when targeted at high-prevalence birth cohorts, the so-called baby-boomers (1945–1965). In response to this, CDC has recommended these cohorts to be offered screening [87]. In Europe, a French study from 2003 suggested HCV screening for the general population could be cost-effective [48]. More evidence on general population HCV screening needed is needed for European countries, especially for those with a relatively high prevalence.

#### Limitations

The main limitation of our review is regarding the comparability of the estimates found. First this is limited since different laboratory tests were used, particularly for HCV where antibody assay validity has improved in recent years. Second, prevalence estimates were not always standardized by age and sex. Lastly, the definition and sampling of the high risk population groups are likely to influence prevalence estimates found both for these groups as for the general population. A limitation of the cost-effectiveness studies is that most analyses used Markov modeling, necessary since the disease outcomes of chronic HBV and HCV infection take several decades to develop. When not accounting for co-morbidities such as excess of alcohol intake, these models can overestimate the effects of screening and treatment by having too optimistic assumptions about life expectancy. On the other hand, effects of screening are underestimated since these models do not allow quantifying the effect of reduced transmission by lowering viral load due to antiviral treatment and potential behavior change. Since these effects can be considerable [13-15], dynamic models assessing the effects of screening and treatment need to be developed. This is likely to be of particular relevance for population subgroups where not only the prevalence, but also the incidence, is increased compared to the general population, such as MSM (HBV) and PWID (HCV). A recent study from Martin et al. did include indirect effects of treating HCV infections in PWID. It was not included in our review since it assessed cost-effectiveness of treatment rather than of screening and treatment [88].

Lastly, methods, assumptions, and quality varied between studies, making it difficult to compare results and limiting the possibilities of carrying out meta-analyses. Guidelines such as those developed for economic analyses of vaccination programmes may be helpful to improve the quality of studies [89,90].

## Conclusions

Available data suggest a wide variation in prevalence of chronic HBV and HCV infection between countries in Europe. Countries in the south and east of the European Union and in Turkey have a much higher prevalence for chronic HBV and HCV than countries in northwestern Europe. For the majority of countries data on the general population prevalence of HBV or HCV are lacking. Within countries, the prevalence of HBsAg and anti-HCV-Ab among PWID, MSM, and migrants is generally much higher than the general population prevalence. Considerable health benefits can be gained cost-effectively by anti-HCV-Ab screening of PWID. HBsAg screening of pregnant women and migrants is also very likely cost-effective. Appraisals of the evidence for screening the general population in mid- and highly endemic countries in Europe and of combined HBV/HCV screening are needed. Future cost-effectiveness analyses may need to take the effect of antiviral treatment on preventing HBV and HCV transmission into account.

## Ethics statement

An ethics statement was not required for this work.

## Endnotes

All 27 EU member states, 4 EEA/EFTA countries (Norway, Iceland, Liechtenstein and Switzerland) and 3 EU enlargement countries (Croatia, the former Yugoslav Republic of Macedonia and Turkey).

## Competing interests

The authors declare that they have no competing interests.

## Authors’ contributions

SH, IV, ML and MS designed the study. SH and IV carried out the systematic review and wrote the manuscript. LW provided and interpreted the data on PWID. TL assisted with the economic analyses. All authors contributed to interpreting the data and writing the manuscript. All authors read and approved the final manuscript.

## Pre-publication history

The pre-publication history for this paper can be accessed here:

http://www.biomedcentral.com/1471-2334/13/181/prepub

## Supplementary Material

Additional file 1 **Search strategy. **S1. 1 Prevalence studies, General population, 34 countries European region, Prevalence in 5 specific population subgroups, 34 countries European Region, S1. 2 Cost-effectiveness studies. Click here for file

Additional file 2 **PRISMA flow diagrams **[114]**.** S2.1 Systematic review of seroprevalence of HBsAg and anti-HCV-Ab, S2. 2 Systematic review of cost-effectiveness of screening for chronic HBV and HCV infection. Click here for file

Additional file 3 **HBsAg and anti-HCV-Ab prevalence estimates combined and in population subgroups, by country, European neighbourhood. Figure S2.** Summary of HBsAg and anti-HCV-Ab prevalence profiles in Europe, 2000–2009. **Figure S3.** 1a First-time blood donors: HBsAg prevalence (%) by country, Europe, 2000–2009. **Figure S3.** 1b First-time blood donors: anti-HCV-Ab prevalence (%) by country, Europe, 2000–2009. **Figure S3.** 2a Pregnant women: HBsAg prevalence (%) by country, Europe, 2000–2009. **Figure S3.** 2b Pregnant women: anti-HCV-Ab prevalence (%) by country, Europe, 2000–2009. **Figure S3.** 3a People who inject drugs (PWID): HBsAg prevalence (%) by country, Europe, 2000–2009. **Figure S3.** 3b PWID: anti-HCV-Ab prevalence (%)by country, Europe, 2000–2009. Click here for file

Additional file 4: Table S3 1a First-time blood donors: HBsAg prevalence (%) by country, Europe, 2000–2009. **Table S3.** 1b First-time blood donors: anti-HCV-Ab prevalence (%) by country, Europe, 2000–2009. **Table S3.** 2a Pregnant women: HBsAg prevalence (%) by country, Europe, 2000–2009. **Table S3.** 2b Pregnant women: anti-HCV-Ab prevalence (%) by country, Europe, 2000–2009. **Table S3.** 3a PWID: HBsAg prevalence (%) by country, Europe, 2000–2009, **Table S3.** 3b PWID: anti-HCV-Ab prevalence (%) by country, Europe, 2000–2009. **Table S3.** 4a Migrants: HBsAg prevalence (%) by country of residence, Europe, 2000–2009 **Table S3.** 4b Migrants: anti-HCV-Ab prevalence (%) by country of residence, Europe, 2000–2009. Click here for file
