# Supplementary material for: Infection with hepatitis B and C virus in Europe: a systematic review of prevalence and cost-effectiveness of screening
Source: BMC Infect Dis. 2013 Apr 18;13:181. doi: 10.1186/1471-2334-13-181 (PMC3716892; doi:10.1186/1471-2334-13-181)
Supplement: Additional file 1 — Search strategy. S1. 1 Prevalence studies, General population, 34 countries European region, Prevalence in 5 specific population subgroups, 34 countries European Region, S1. 2 Cost-effectiveness studies. [file 1471-2334-13-181-S1.doc]

**Infection with hepatitis B and C virus in Europe: a systematic review of prevalence and cost-effectiveness of screening: Supporting information**

S. Hahné et al.

**Supporting information 1: Search strategy**

**S1.1 Prevalence studies**

**General population, 34 countries European region**

| **Ovid-MEDLINE** | |
| --- | --- |
|  |  |
| 1 | (hepatitis B or hbv or hepatitis c or hcv].ti. |
| 2 | exp hepatitis b/ or exp hepatitis c/ |
| 3 | 1 or 2 |
| 4 | (prevalence or seroprevalence or seroepidemiolog* or serologic* markers or serology or residual sera or hbsag or hbs ag or hepatitis b surface antigen* or hbv surface antigen* or anti-hcv or anti hcv or hcv rna or carrier*].tw. |
| 5 | prevalence/ or seroepidemiological studies/ or hepatitis b surface antigens/ or hepatitis c antibodies/ or carrier state/ |
| 6 | 3 and (4 or 5] |
| 7 | (population or community or child* or adolesc* or adults or elder* or older or surveillance or serosurveillance or survey*].tw. |
| 8 | population surveillance/ or health surveys/ |
| 9 | 6 and (7 or 8] |
| 10 | ("european populations" or europ* or iceland or norway or sweden or finland or denmark or "great britain" or england or scotland or wales or ireland or netherlands or belgium or france or luxemburg or spain or portugal or italy or switzerland or austria or germany or poland or hungary or czech or croatia or slovakia or slovenia or romania or bulgaria or lithuania or latvia or estonia or estland or greece or turkey or macedonia or cyprus or malta].tw. |
| 11 | exp europe/ or european union/ |
| 12 | 9 and (10 or 11] |
| 13 | limit 12 to yr=2000-2009 |
| 14 | 13 and english.lg. |

| **DIMDI** (sbas me90;em90;is74;rd=01.01.2000-27.07.2009] | |
| --- | --- |
| 1 | c=me90; em90; is74 |
| 2 | s=ft=(hepatitis b;hbv;hepatitis c;hcv]/ti |
| 3 | ct d (hepatitis b;hepatitis c] |
| 4 | 2 or 3 |
| 5 | ft=(prevalence;seroprevalence;seroepidemiolog?;serologic |
|  | ? markers;serology;residual sera;hbsag;hbs ag;hepatitis |
|  | b surface antigen?;hbv surface antigen?;anti-hcv;anti |
|  | hcv;hcv rna;carrier?]/(ti;ab] |
| 6 | ct=(prevalence;seroepidemiological |
|  | studies;seroepidemiology;hepatitis b surface |
|  | antigens;hepatitis b surface antigen;hepatitis c |
|  | antibodies;carrier state] |
| 7 | 4 and (5 or 6] |
| 8 | ft=(population;community;child?;adolesc?;adults;elder?; |
|  | older;surveillance;serosurveillance;survey?]/(ti;ab] |
| 9 | ct=(population surveillance;population |
|  | research;population exposure;health surveys;health |
|  | survey] |
| 10 | 7 and (8 or 9] |
| 11 | ft=(european populations;europ?;iceland;norway; sweden;finland; denmark; great britain; england;scotland;wales;ireland; netherlands; belgium; france;luxemburg;spain;portugal;italy;switzerland;austria; |
|  |
|  |
|  | ;germany]/(ti;ab] |
| 12 | ft=(poland;hungary;czech;croatia;slovakia;slovenia;romania; |
|  | bulgaria;lithuania;latvia;estonia;estland;greece;turk |
|  | ey;macedonia;cyprus;malta]/(ti;ab] |
| 13 | ct d europe or ct=(european union;european economic |
|  | community] |
| 14 | 10 and (11 or 12 or 13] |
| 15 | 14 and py>1999 |
| 16 | 15 and la=english |
| 17 | check duplicates: unique in s=16 |
| 18 | 17 and base=me90 |
| 19 | 17 not 18 |

**Prevalence in 5 specific population subgroups, 34 countries European Region**

| **Ovid-Medline** | |
| --- | --- |
| 1 | (hepatitis B or hbv or hepatitis c or hcv].ti. |
| 2 | exp hepatitis b/ or exp hepatitis c/ |
| 3 | 1 or 2 |
| 4 | (prevalence or seroprevalence or seroepidemiolog* or serologic* markers or serology or residual sera or hbsag or hbs ag or hepatitis b surface antigen* or hbv surface antigen* or anti-hcv or anti hcv or hcv rna or carrier*].tw. |
| 5 | prevalence/ or seroepidemiological studies/ or hepatitis b surface antigens/ or hepatitis c antibodies/ or carrier state/ |
| 6 | 3 and (4 or 5] |
| 7 | (blood donor* or blood-donor* or idu or injecting drug users or intravenous drug users or substance abuse* or drug abuse* or drug users].tw. |
| 8 | (msm or (men adj3 sex adj3 men] or homosex* or (homo adj3 sexual] or gay men or migrant* or immigrant* or minorit* or pregnan* or antenatal or prenatal].tw. |
| 9 | blood donors/ or intravenous substance abuse/ or drug users/ or male homosexuality/ or "transients and migrants"/ or "emigrants and immigrants"/ or minority groups/ or pregnancy/ |
| 10 | 6 and (7 or 8 or 9] |
| 11 | ("european populations" or europ* or iceland or norway or sweden or finland or denmark or "great britain" or england or scotland or wales or ireland or netherlands or belgium or france or luxemburg or spain or portugal or italy or switzerland or austria or germany or poland or hungary or czech or croatia or slovakia or slovenia or romania or bulgaria or lithuania or latvia or estonia or estland or greece or turkey or macedonia or cyprus or malta].tw. |
| 12 | exp europe/ or european union/ |
| 13 | 10 and (11 or 12] |
| 14 | limit 13 to yr=2000-2009 |
| 15 | 14 and english.lg. |

| **DIMDI** (sbas me90;em90;is74;rd=01.01.2000-27.07.2009] | |
| --- | --- |
| 1 | c=1 me90; em90; is74 |
| 2 | s=2 ft=(hepatitis b;hbv;hepatitis c;hcv]/ti |
| 3 | ct d (hepatitis b;hepatitis c] |
| 4 | 2 or 3 |
| 5 | ft=(prevalence;seroprevalence;seroepidemiolog?;serologic |
|  | ? markers;serology;residual sera;hbsag;hbs ag;hepatitis |
|  | b surface antigen?;hbv surface antigen?;anti-hcv;anti |
|  | hcv;hcv rna;carrier?]/(ti;ab] |
| 6 | ct=(prevalence;seroepidemiological |
|  | studies;seroepidemiology;hepatitis b surface |
|  | antigens;hepatitis b surface antigen;hepatitis c |
|  | antibodies;carrier state] |
| 7 | 4 and (5 or 6] |
| 8 | ft=(blood donor?;blood-donor?;idu;injecting drug |
|  | users;intravenous drug users;substance abuse?;drug |
|  | abuse?;drug users]/(ti;ab] |
| 9 | ft=(msm;men # # # sex # # # men;homosex?;homo # # #  sexual;gay men;migrant?;immigrant?; minorit?;pregnan?;antenatal;pren atal]/(ti;ab] |
|  |
| 10 | ct=(blood donors;blood donor;intravenous substance |
|  | abuse;intravenous drug abuse;drug users;drug use;drug |
|  | abuse;male homosexuality;homosexuality;transients and |
|  | migrants;emigrants and immigrants;immigrant;minority |
|  | groups;minority group;pregnancy] |
| 11 | 7 and (8 or 9 or 10] |
| 12 | ft=(european populations;europ?;iceland; norway;sweden;finland;denmark; great  britain;england;scotland;wales;ireland; netherlands;belgi um;france;luxemburg;spain;portugal;italy;switzerland;aus tria; germany]/(ti;ab] |
|  |
|  |
| 13 | ft=(poland;hungary;czech;croatia;slovakia;slovenia;roman ia; bulgaria;lithuania;latvia;estonia;estland;greece;turkey; macedonia;cyprus;malta]/(ti;ab] |
|  |
|  |
| 14 | ct d europe or ct=(european union;european economic |
|  | community] |
| 15 | 11 and (12 or 13 or 14] |
| 16 | 15 and py>1999 |
| 17 | 16 and la=english |
| 18 | check duplicates: unique in s=17 |
| 19 | 18 and base=me90 |
| 20 | 18 not 19 |

S1.2 Cost-effectiveness studies

| **Ovid MEDLINE(R], Ovid MEDLINE(R] In-Process** | |
| --- | --- |
| 1 | (hepatitis B or hbv or hepatitis c or hcv or hbsag or hbs ag or hcv-rna or anti-hcv].ti. |
| 2 | exp hepatitis b/ or exp hepatitis c/ or hepatitis b surface antigens/ or hepatitis c antibodies/ |
| 3 | 1 or 2 |
| 4 | (screening or testing].tw. |
| 5 | mass screening/ |
| 6 | 3 and (4 or 5] |
| 7 | cost-benefit analysis/ or "costs and cost analysis"/ or mass screening/ec or quality-adjusted life years/ |
| 8 | (cost benefit* or cost effect* or cost utilit* or cost efficien* or econom* or quality adjusted or disability adjusted or qaly* or daly* or icer].ti. |
| 9 | 6 and (7 or 8] |
| 10 | (blood donors or blood recipients or blood donations or transfusion* or posttransfusion or donor* or donation* or blood screening or blood supply or blood product* or plasma product* or plasma or postdonation or coagulation factor concentrates or blood bank or transplantation or hepatocellular carcinoma or HCC].ti. |
| 11 | 9 not 10 |
| 12 | 11 and english.lg. |
| 13 | limit 12 to yr=1990-2011 |
| 14 | remove duplicates from 13 |

| **Scopus** |
| --- |
| (((TITLE((hepatitis-b] OR hbv OR (hepatitis-c] OR hcv OR hbsag OR (hbs-ag] OR (hcv-rna] OR (anti-hcv]] OR KEY((hepatitis-b] OR hbv OR (hepatitis-c] OR hcv OR hbsag OR (hbs-ag] OR (hcv-rna] OR (anti-hcv]]] AND (TITLE(screening OR testing] OR KEY(screening OR testing]] AND (TITLE((cost-benefit*] OR (cost-effect*] OR (cost-utilit*] OR (cost-efficien*] OR econom* OR (quality-adjusted] OR (disability-adjusted] OR qaly* OR daly* OR icer] OR KEY((cost-benefit*] OR (cost-effect*] OR (cost-utilit*] OR (cost-efficien*] OR econom* OR (quality-adjusted] OR (disability-adjusted] OR qaly* OR daly* OR icer]]] AND NOT (TITLE((blood-donors] OR (blood-recipients] OR (blood-donations] OR transfusion* OR posttransfusion OR donor* OR donation* OR (blood-screening] OR (blood-supply] OR (blood-product*] OR (plasma-product*] OR plasma OR postdonation OR (coagulation-factor-concentrates] OR (blood-bank] OR transplantation OR (hepatocellular-carcinoma] or HCC]]] AND (LANGUAGE(english] AND PUBYEAR AFT 1989] |
