# Supplementary material for: Infection with hepatitis B and C virus in Europe: a systematic review of prevalence and cost-effectiveness of screening
Source: BMC Infect Dis. 2013 Apr 18;13:181. doi: 10.1186/1471-2334-13-181 (PMC3716892; doi:10.1186/1471-2334-13-181)
Supplement: Additional file 2 — PRISMA flow diagrams [114]. S2.1 Systematic review of seroprevalence of HBsAg and anti-HCV-Ab, S2. 2 Systematic review of cost-effectiveness of screening for chronic HBV and HCV infection. [file 1471-2334-13-181-S2.doc]

**Additional file 2: PRISMA flow diagrams^116^**

**S2.1 Systematic review of seroprevalence of HBsAg and anti-HCV-Ab**

Records identified through database searching
(n = 1759)

Additional records identified through other sources
(n = 8)

Records after duplicates removed
(n = 1767)

Records screened
(n =1767)

Records excluded
(n = 1523)

Full-text articles assessed for eligibility
(n = 244)

Full-text articles excluded, with reasons
(n =120)

Studies included in qualitative synthesis
(n = 124)

Studies included in quantitative synthesis
(n = 81)

**S2.2 Systematic review of cost-effectiveness of screening for chronic HBV and HCV infection**

Records identified through database searching
(n = 468)

Additional records identified through other sources
(n = 3)

Records after duplicates removed
(n = 471 )

Records screened
(n =471)

Records excluded
(n = 427)

Full-text articles assessed for eligibility
(n = 44)

Full-text articles excluded, with reasons
(n =15)

Studies included in qualitative synthesis
(n = 29)
