# Supplementary material for: Infection with hepatitis B and C virus in Europe: a systematic review of prevalence and cost-effectiveness of screening
Source: BMC Infect Dis. 2013 Apr 18;13:181. doi: 10.1186/1471-2334-13-181 (PMC3716892; doi:10.1186/1471-2334-13-181)
Supplement: Additional file 3 — HBsAg and anti-HCV-Ab prevalence estimates combined and in population subgroups, by country, European neighbourhood. Figure S2. Summary of HBsAg and anti-HCV-Ab prevalence profiles in Europe, 2000–2009. Figure S3. 1a First-time blood donors: HBsAg prevalence (%) by country, Europe, 2000–2009. Figure S3. 1b First-time blood donors: anti-HCV-Ab prevalence (%) by country, Europe, 2000–2009. Figure S3. 2a Pregnant women: HBsAg prevalence (%) by country, Europe, 2000–2009. Figure S3. 2b Pregnant women: anti-HCV-Ab prevalence (%) by country, Europe, 2000–2009. Figure S3. 3a People who inject drugs (PWID): HBsAg prevalence (%) by country, Europe, 2000–2009. Figure S3. 3b PWID: anti-HCV-Ab prevalence (%)by country, Europe, 2000–2009. [file 1471-2334-13-181-S3.doc]

**Additional file 3: Figures: HBsAg and anti-HCV-Ab prevalence estimates combined and in population subgroups, by country, European neighbourhood.**

**Figure S2. Summary of HBsAg and anti-HCV-Ab prevalence profiles in Europe, 2000-2009.**

**
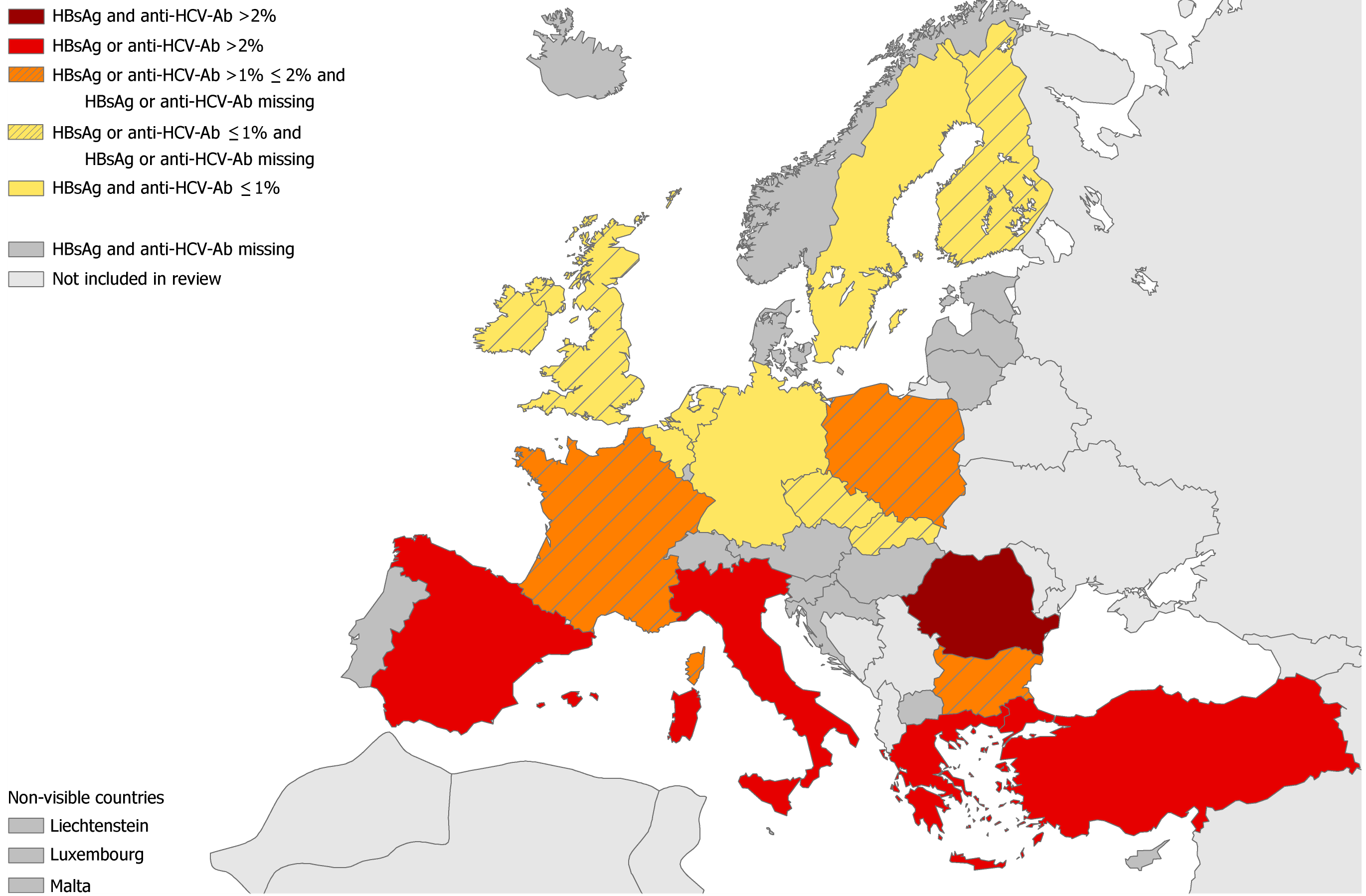
**

**Figure S3.1a First-time blood donors: HBsAg prevalence (%) by country, Europe, 2000-2009.**

**Figure S3.1b First-time blood donors: anti-HCV-Ab prevalence (%) by country, Europe, 2000- 2009.**

**Figure S3.2a Pregnant women: HBsAg prevalence (%) by country, Europe, 2000-2009.**

**Figure S3.2b Pregnant women: anti-HCV-Ab prevalence (%) by country, Europe, 2000-2009.**

**Figure S3.3a People who inject drugs (PWID): HBsAg prevalence (%) by country, Europe, 2000-2009.**

***
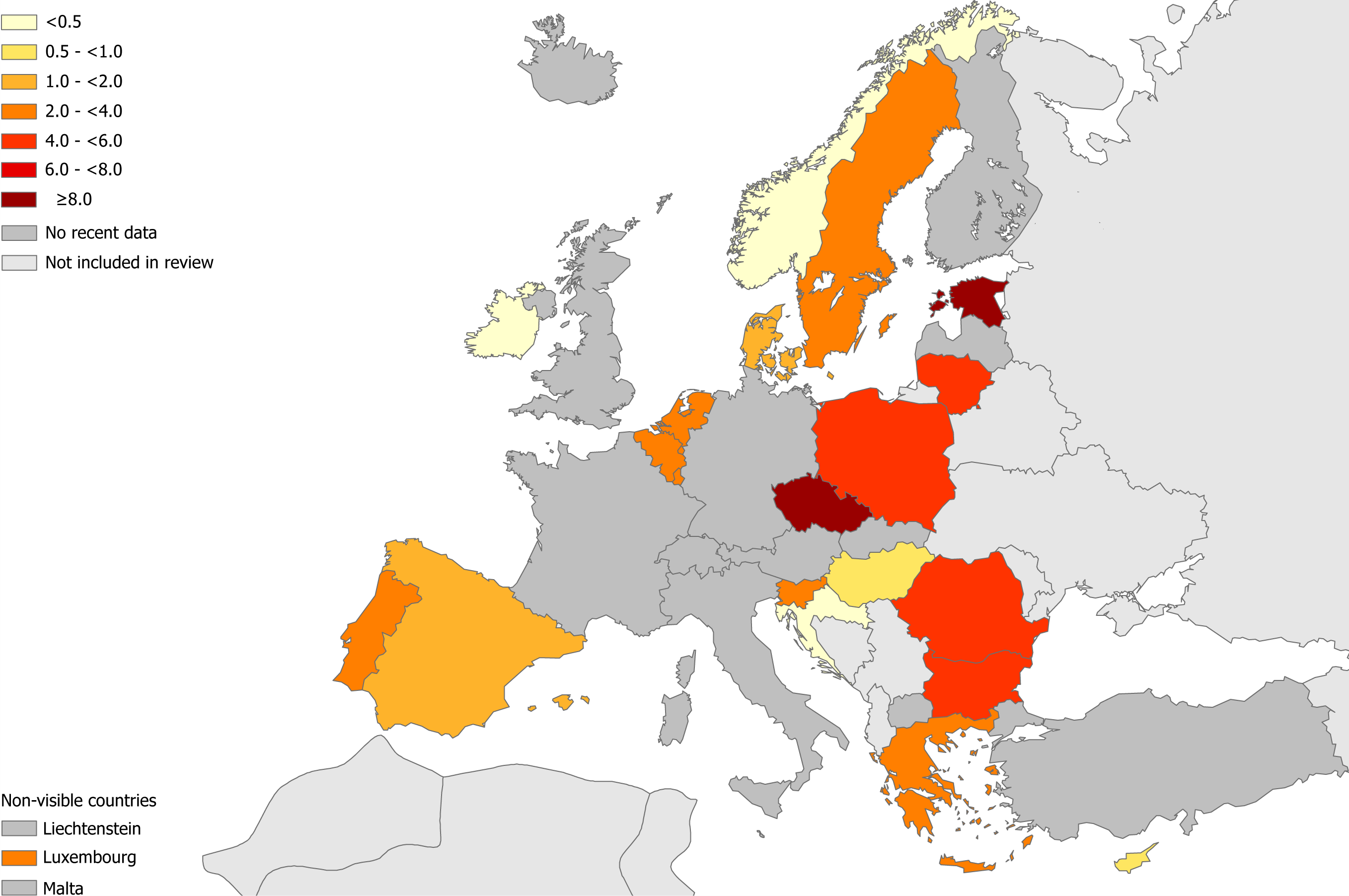
***

**Figure S3.3b PWID: anti-HCV-Ab prevalence (%) by country, Europe, 2000-2009.**

**
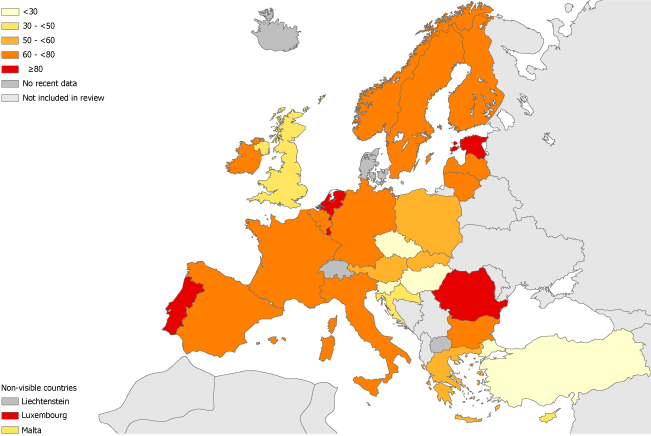
**
