# Supplementary material for: Infection with hepatitis B and C virus in Europe: a systematic review of prevalence and cost-effectiveness of screening
Source: BMC Infect Dis. 2013 Apr 18;13:181. doi: 10.1186/1471-2334-13-181 (PMC3716892; doi:10.1186/1471-2334-13-181)
Supplement: Additional file 4: Table S3 — 1a First-time blood donors: HBsAg prevalence (%) by country, Europe, 2000–2009. Table S3. 1b First-time blood donors: anti-HCV-Ab prevalence (%) by country, Europe, 2000–2009. Table S3. 2a Pregnant women: HBsAg prevalence (%) by country, Europe, 2000–2009. Table S3. 2b Pregnant women: anti-HCV-Ab prevalence (%) by country, Europe, 2000–2009. Table S3. 3a PWID: HBsAg prevalence (%) by country, Europe, 2000–2009, Table S3. 3b PWID: anti-HCV-Ab prevalence (%) by country, Europe, 2000–2009. Table S3. 4a Migrants: HBsAg prevalence (%) by country of residence, Europe, 2000–2009 Table S3. 4b Migrants: anti-HCV-Ab prevalence (%) by country of residence, Europe, 2000–2009. [file 1471-2334-13-181-S4.doc]

**Table S3.1a First-time blood donors: HBsAg prevalence (%) by country, Europe, 2000-2009.**

| **Country (Reference)** | **Period** | **Prevalence (%)** |
| --- | --- | --- |
| Belgium (31) | 2005 | 0.06 |
| Bulgaria (31) | 2005 | 5.2 |
| Croatia (31) | 2005 | 0.2 |
| Cyprus (117) | Not reported | 3.0 |
| Czech Republic (31) | 2005 | 0.07 |
| Finland (31) | 2005 | 0.04 |
| France (31) | 2005 | 0.1 |
| Germany (118) | 1997-2002 | 0.2 |
| Greece (119) | 1995-1997 | 0.9 |
| Hungary (31) | 2005 | 0.00 |
| Ireland (31) | 2005 | 0.02 |
| Italy (120) | 2005 | 0.4 |
| Lithuania (121) | 2005-2006 | 1.7 |
| Luxembourg (31) | 2005 | 0.1 |
| Netherlands (31) | 2005 | 0.09 |
| Norway (31) | 2005 | 0.02 |
| Poland (122) | 1998-2000 | 0.9 |
| Romania (31) | 2005 | 4.3 |
| Slovakia (31) | 2005 | 0.2 |
| Slovenia (31) | 2005 | 0.09 |
| Spain (31) | 2005 | 0.1 |
| Sweden (31) | 2005 | 0.06 |
| Switzerland (31) | 2005 | 0.1 |
| United Kingdom (31) | 2005 | 0.04 |

**Table S3.1b First-time blood donors: anti-HCV-Ab prevalence (%) by country, Europe, 2000-2009.**

| **Country (Reference)** | **Period** | **Prevalence (%)** |
| --- | --- | --- |
| Belgium (31) | 2005 | 0.06 |
| Bulgaria (31) | 2005 | 0.9 |
| Croatia (31) | 2005 | 0.06 |
| Cyprus (117) | 2005 | 0.5 |
| Czech Republic (31) | 2005 | 0.1 |
| Finland (31) | 2005 | 0.04 |
| France (31) | 2005 | 0.06 |
| Germany (31) | 2005 | 0.08 |
| Germany (118) | 1997-2002 | 0.1 |
| Greece (31) | 2005 | 0.6 |
| Hungary (31) | 2005 | 0.3 |
| Ireland (31) | 2005 | 0.02 |
| Lithuania (121) | 2005-2006 | 1.7 |
| Luxembourg (31) | 2005 | 0.06 |
| Netherlands (31) | 2005 | 0.03 |
| Norway (31) | 2005 | 0.06 |
| Poland (122) | 1998-2000 | 0.6 |
| Romania (31) | 2005 | 3.3 |
| Slovakia (31) | 2005 | 0.06 |
| Slovenia (31) | 2005 | 0.02 |
| Spain (123) | 1999-2001 | 0.2 |
| Sweden (31) | 2005 | 0.1 |
| Switzerland (31) | 2005 | 0.08 |
| United Kingdom (31) | 2005 | 0.04 |

**Table S3.2a Pregnant women: HBsAg prevalence (%) by country, Europe, 2000-2009.**

| **Country (Reference)** | **Period** | **Area** | **Region** | **N** | **Prevalence (%)** | **95% CI** |
| --- | --- | --- | --- | --- | --- | --- |
| Denmark (124) | 2005-2006 | Nationwide |  | 29,708 | 0.3 |  |
| France (125) | 1984-1998 | Regional | Limoges | 22,859 | 0.7 |  |
| Germany (126) | 1996-2005 | Regional | Heidelberg | 5,518 | 1.6 |  |
| Greece (127) | 2003 | Nationwide |  | 3,384 | 2.9 | 2.3-3.4 |
| Ireland (128) | 1998-2000 | Regional | Dublin | 16,222 | 0.4 |  |
| Italy (129) | 2001 | Nationwide |  | 10,881 | 1.7 | 1.4-1.9 |
| Netherlands (130) | 1993-1998 | Regional | Amsterdam | 56,756 | 1.2 |  |
| Slovakia (131) | 2000-2004 | Regional | Bratislava | 90 | 4.4 |  |
| Spain (132) | 2004 | Regional | Catalonia | 1,534 | 0.1 | 0.0-0.3 |
| Switzerland (133) | 2001 | Regional | Basel | 1,503 | 1.2 | 0.7-1.8 |
| United Kingdom (134) | 2002 | Regional | London | 110,621 | 1.0 |  |

**Table S3.2b Pregnant women: anti-HCV-Ab prevalence (%) by country**, Europe, 2000-2009.

| **Country (Reference)** | **Period** | **Area** | **Region** | **N** | **Prevalence (%)** | **95% CI** |
| --- | --- | --- | --- | --- | --- | --- |
| Germany (135) | 1992-1996 | regional | Munich | 3,712 | 0.9 |  |
| Greece (136) | 1994-2002 | regional | Athens (Piraeus) | 5,497 | 0.8 |  |
| Greece (137) | 1996-1997 | regional | North | 2,408 | 2.0 |  |
| Italy (138) | 1996 | regional | North | 2,059 | 1.9 |  |
| Italy (139) | 1995-1998 | regional | North | 15,25 | 2.4 |  |
| Italy (140) | 1996-2001 | regional | North | 13,025 | 0.8 |  |
| Slovakia (131) | 2000-2004 | regional | Bratislava | 90 | 0.0 |  |
| Switzerland (141) | 1990-1991 | nationwide |  | 9,057 | 0.7 |  |
| United Kingdom (142) | 1997-1998 | nationwide | | 126,009 | 0.2 | 0.1-0.3 |
| United Kingdom (143) | 1996 | regional | Northern and Yorkshire | 16,675 | 0.2 | 0.1-0.3 |
| United Kingdom (143) | 1996 | regional | London | 25,94 | 0.4 | 0.3-0.5 |
| United Kingdom (144) | 1997-1999 | regional | London | 4,729 | 0.8 | 0.6-1.0 |
| United Kingdom (145) | 1997 | regional | Scotland | 3,548 | 0.6 | 0.4-1.0 |
| United Kingdom (146) | 2000 | regional | Scotland | 30,259 | 0.3 |  |

**Table S3.3a PWID: HBsAg prevalence (%) by country, Europe, 2000-2009.**

| **Country* (Reference)** | **Period** | **Area** | **Region** | **N** | **Prevalence (%)** | **95% CI*** |
| --- | --- | --- | --- | --- | --- | --- |
| Belgium (32) | 2008-2009 | Subnational | Antwerp, Flemish community | 434 | 3.5 | 2.0-5.6 |
| Bulgaria (32) | 2009 | Subnational | Sofia | 941 | 5.8 | 4.4-7.5 |
| Croatia (32) | 2007 | National |  | 200 | 0.5 | 0.0-2.8 |
| Cyprus (32) | 2009 | National | - | 115 | 0.9 | 0.0-4.8 |
| Czech Rep. (33) | 2010 | Not reported |  | 575 | 15·1 | 12.3-18.3 |
| Denmark (33) | 2007 | Subnational | Funen | 239 | 1·3 | 0.3-3.6 |
| Estonia (33) | 2004 | Subnational | Tallinn | 155 | 21·3 | 15.1-28.6 |
| Greece (32) | 2009 | National |  | 1814 | 2.6 | 1.9-3.4 |
| Hungary (32) | 2009 | National | - | 676 | 0.7 | 0.2-1.7 |
| Ireland (32) | 2003 | Subnational | Dublin | 63 | 0.0 | 0.0-5.7 |
| Lithuania (32) | 2005-2006 | Subnational | Alytus city, Vilnius | 517 | 4.4 | 2.8-6.6 |
| Luxembourg (32) | 2005 | National | - | 255 | 3.9 | 1.9-7.1 |
| Netherlands (32) | 2000 | Subnational | The Hague | 199 | 3.0 | 1.1-6.5 |
| Norway (32) | 2009 | Subnational | Oslo | 179 | 0.0 | 0.0-2.0 |
| Poland (32) | 2002-2009 | Subnational | Eight cities | 952 | 4.2 | 3.0-5.7 |
| Portugal (32) | 2009 | National | - | 838 | 2.9 | 1.8-4.2 |
| Romania (32) | 2009 | Subnational | Bucharest | 447 | 4.7 | 2.9-7.1 |
| Slovenia (32) | 2002 | National | - | 564 | 3.4 | 2.0-5.2 |
| Spain (33) | 2006 | Subnational | Barcelona | 166 | 1·8 | 0.4-5.2 |
| Sweden (33) | 2006 | Subnational | Stockholm | 310 | 2·6 | 1.1-5.0 |

* CI Confidence interval

No estimate of HBsAg prevalence for PWID was available for Austria, Finland, the FYR Macedonia, France, Germany, Iceland, Italy, Latvia, Liechtenstein, Malta, Slovakia, Switzerland, Turkey and the United Kingdom.

For EMCDDA data (32) original sources are available online: <http://www.emcdda.europa.eu/stats11/inftab0>

**Table S3.3b PWID: anti-HCV-Ab prevalence (%) by country**, Europe, 2000-2009.

| **Country (Reference)** | **Period** | **Area** | **Region** | **N** | **Prevalence**  **(%)** | **95% CI*** |
| --- | --- | --- | --- | --- | --- | --- |
| Austria (32) | 2009 | National |  | 511 | 53.2 | 48.8 - 57.6 |
| Belgium (32) | 2008-2009 | Subnational | Antwerp, Flemish community | 454 | 68.3 | 63.9 - 72.5 |
| Bulgaria (32) | 2009 | Subnational | Sofia | 955 | 61.2 | 58.0 - 64.3 |
| Croatia (32) | 2007 | National | - | 200 | 44.0 | 37.0 - 51.2 |
| Cyprus (32) | 2009 | National | - | 116 | 46.6 | 37.2 - 56.1 |
| Czech rep. (32) | 2009 | National | - | 353 | 22.4 | 18.1 - 27.1 |
| Estonia (32) | 2002 | Subnational | Ida-Viru, Tallinn | 100 | 90.0 | 82.4 - 95.1 |
| Finland (32) | 2009 | National | - | 682 | 60.5 | 56.8 - 64.3 |
| France (32) | 2006 | Subnational | Five cities | 362 | 41.7 | 36.6 - 47.0 |
| Germany (33) | 2001-2003 | Subnational | Warstein | 1512 | 75·0 | 72.7 - 77.2 |
| Greece (32) | 2009 | National |  | 1751 | 55.0 | 52.6 - 57.4 |
| Hungary (32) | 2009 | National | - | 667 | 24.4 | 21.2 - 27.9 |
| Ireland (32) | 2003 | Subnational | Dublin | 65 | 72.3 | 60.0 - 82.7 |
| Italy (32) | 2000 | National | - | 628 | 72.9 | 69.3 - 76.4 |
| Latvia (32) | 2007 | Subnational | Riga | 406 | 74.4 | 69.9 - 78.6 |
| Lithuania (32) | 2000 | National | - | 693 | 79.0 | 75.7 - 81.9 |
| Luxembourg (32) | 2005 | National | - | 268 | 81.3 | 76.2 - 85.8 |
| Malta (32) | 2009 | National | - | 121 | 30.6 | 22.5 - 39.6 |
| Netherlands (32) | 2008 | Subnational | Rotterdam | 65 | 86.2 | 75.3 - 93.5 |
| Norway (32) | 2009 | National | - | 3972 | 72.9 | 71.5 - 74.3 |
| Poland (32) | 2009 | Subnational | Eight cities | 950 | 59.2 | 56.0 - 62.3 |
| Portugal (32) | 2009 | National | - | 895 | 83.1 | 80.5 - 85.5 |
| Romania (32) | 2009 | Subnational | Bucharest | 449 | 82.9 | 79.0 - 86.2 |
| Slovakia (32) | 2009 | Subnational | Bratislava | 98 | 50.0 | 39.7 - 60.3 |
| Slovenia (32) | 2009 | National | - | 401 | 23.4 | 19.4 - 27.9 |
| Spain (32) | 2001-2003 | Subnational | Barcelona, Madrid, Seville | 912 | 77.2 | 74.3 - 80.0 |
| Sweden (32) | 2008, 2009 | Subnational | Stockholm county, Sotckholm, Gothenburg | 683 | 75.5 | 72.2 - 78.7 |
| Turkey (32) | 2008 | Subnational | Gaziantep | 168 | 5.3 | 2.5 - 9.9 |
| UK (32) | 2008, 2009 | Subnational | England & Wales, Northern Ireland, Scotland | 3112 | 45.3 | 43.6 - 47.1 |

* CI Confidence interval

No estimate of anti-HCV prevalence for PWID was available for Denmark, the FYR Macedonia, Iceland, Liechtenstein and Switzerland.

For EMCDDA data (32) original sources are available online: <http://www.emcdda.europa.eu/stats11/inftab0>

**Table S3.4a Migrants: HBsAg prevalence (%) by country of residence**, Europe, 2000-2009.

| **Country (Reference)** | **Study period** | **Country of birth / ethnicity** | **Status** | **N** | **Prevalence**  **(%)** | **Remark** |
| --- | --- | --- | --- | --- | --- | --- |
| Greece (147) | not reported | Albania | Refugees | 130 | 15.4 |  |
| Italy (148) | 1997 | Albania | Refugees | 670 | 13.6 |  |
| Italy (149) | 2005-2006 | South America | Refugees | 130 | 10.7 |  |
| Italy (150) | 2005 | Africa, Asia | Refugees | 556 | 10.7 |  |
| Italy (151) | 2005 | Sub-Sahara Africa | Undocumented migrants | 182 | 9.3 |  |
| Italy (152) | 2003-2004 | Several countries | Refugees | 890 | 9.3 |  |
| Spain (153) | 2001-2004 | Several countries | Residents | 1,905 | 7.7 |  |
| Italy* (154) | 2000 | Turkey (Kurds) | Refugees | 368 | 6.8 |  |
| United Kingdom (155) | 2000 | Somalia | Residents | 448 | 5.7 |  |
| Greece (156) | 2002 | Roma | Residents | 118 | 4.2 | Children only |
| Italy (157) | 1999 | Kosovo | Refugees | 526 | 2.9 |  |
| Italy* (154) | 2000 | Iraq (Kurds) | Refugees | 637 | 2.2 |  |
| Netherlands (158) | 2004 | Several countries | Residents | 205 | 1.0 |  |

* One publication on 2 migrant groups

**Table S3.4b Migrants: anti-HCV-Ab prevalence (%) by country of residence**, Europe, 2000-2009.

| **Country** | **Study period** | **Country of birth / ethnicity** | **Status** | **N** | **Prevalence**  **(%)** | **Remark** |
| --- | --- | --- | --- | --- | --- | --- |
| Hungary (159) | 2004 | Roma | Residents | 64 | 23.4 |  |
| Spain (153) | 2001-2004 | Several countries | Residents | 1,848 | 3.1 |  |
| Greece (147) | not reported | Albania | Refugees | 130 | 2.3 |  |
| Italy (151) | 2004-2005 | Sub-Saharan Africa | Undocumented migrants | 182 | 2.2 |  |
| Netherlands (158) | 2004 | Several countries | Residents | 205 | 1.5 |  |
| Italy (160) | 2002-2006 | Several countries | Residents | 120 | 0.8 | Children only |
| Italy (157) | 1999 | Kosovo | Refugees | 526 | 0.7 |  |
| Italy (148) | 1997 | Albania | Refugees | 670 | 0.3 |  |
| Italy* (154) | 2000 | Turkey (Kurds) | Refugees | 368 | 0.1 |  |
| Greece (156) | 2002 | Roma | Residents | 216 | 0.0 | Children only |
| Italy* (154) | 2000 | Iraq (Kurds) | Refugees | 637 | 0.0 |  |
